# Septic Shock Due to Urinary Tract Infection in an Immunosuppressed Patient Prescribed Dapagliflozin

**DOI:** 10.7759/cureus.30552

**Published:** 2022-10-21

**Authors:** Yuto Suetani, Yoh Arita, Yoshinori Iida, Nobuyuki Ogasawara

**Affiliations:** 1 Department of Cardiology, Japan Community Healthcare Organization Osaka Hospital, Osaka, JPN

**Keywords:** female, immunocompromised, urinary tract infections, septic shock, dapagliflozin

## Abstract

Urinary tract infection (UTI) is one of the adverse effects of sodium-glucose cotransporter 2 (SGLT2) inhibitors. We describe a rare case of septic shock due to UTI in an immunosuppressed patient prescribed dapagliflozin. A 69-year-old woman was admitted to our hospital for the treatment of pyelonephritis. She was prescribed immunosuppressive drugs for systemic lupus erythematosus and was newly prescribed dapagliflozin for heart failure two weeks prior. One hour after admission, the patient developed hypotension and was diagnosed with septic shock due to UTI. She was administered norepinephrine, hydrocortisone and meropenem. Afterward, she underwent emergent transurethral lithotomy for her right urinary tract stones. The following clinical course was uneventful, and she was discharged on day 17. She had no recurrence of UTI or exacerbation of heart failure without dapagliflozin administration. This case report emphasizes the importance of considering the possibility of UTIs and cases in which SGLT2 inhibitors should be used. If a patient is female and immunocompromised, dapagliflozin should be prescribed more carefully after considering the increased risk of UTIs.

## Introduction

Sodium-glucose cotransporter 2 (SGLT2) inhibitors have been prescribed worldwide for type 2 diabetes mellitus and heart failure [1,2]. However, several adverse effects of SGLT2 inhibitors have been reported [3]. Urinary tract infection (UTI) is one of the adverse effects of SGLT2 inhibitors. However, there were few reported cases of septic shock due to UTI in a patient prescribed SGLT2 inhibitors. We consider through this case what clinicians should deliberate before prescribing SGLT2 inhibitors.

## Case presentation

A 69-year-old woman presented with fever and chills at night and was transported from home to our hospital by ambulance. On arrival, her body temperature was 39.9°C, blood pressure was 97/60 mmHg, heart rate was 125 beats per minute (bpm), and peripheral oxygen saturation was 96% with 3 L of nasal oxygen inhalation. She had severe tenderness in the right costovertebral angle. The patient presented chronic heart failure due to dilated cardiomyopathy with 36% of left ventricular ejection fraction (LVEF) and New York Heart Association Class III. She was prescribed 10 mg of dapagliflozin, a sodium-glucose cotransporter 2 (SGLT2) inhibitor two weeks prior to admission. She had also been treated for chronic kidney disease (CKD) with stage 3b and systemic lupus erythematosus. She had been prescribed 14 mg of prednisolone and 1 mg of tacrolimus for 33 years. She had a history of bilateral hip prosthesis replacement.

**Figure 1:**
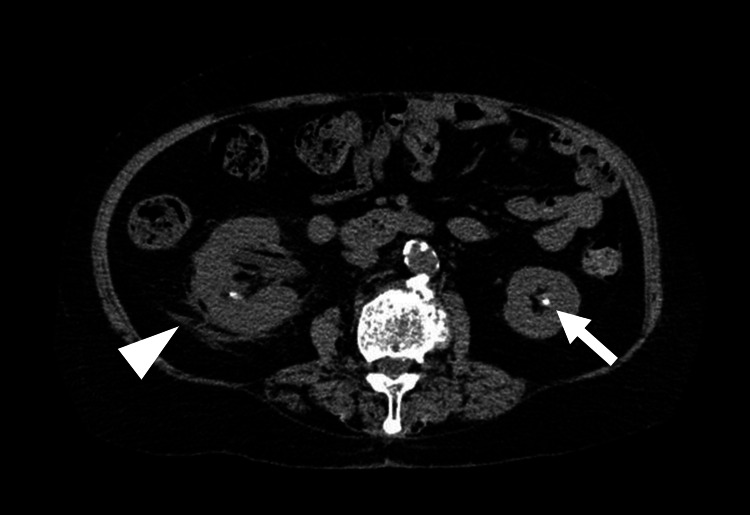
Abdominal computed tomography (CT) on admission CT showing right hydronephrosis, kidney swelling, a right kidney stone, and perirenal fat stranding (arrowhead). It also shows a left kidney stone (arrow).

The patient’s white blood cell count was elevated at 21,800/μL (normal range: 3,300-8,600/μL), and her C-reactive protein (CRP) level was elevated at 2.97 mg/dL (normal range: 0-0.14 mg/dL) (Table 1). Her arterial lactate value was 2.4 mmol/L (normal range: 0-2.0 mmol/L). Her creatinine level was 1.16 mg/dL (normal range: 0.46-0.79 mg/dL), and estimated glomerular filtration rate was 36.1 mL/min/1.73 m^2^ (normal range: >60 mL/min/1.73 m^2^). The Sequential Organ Failure Assessment (SOFA) score was 5. Blood and urine cultures revealed *Escherichia coli* and elevated procalcitonin levels of 2.04 ng/mL (normal range: 0-0.5 ng/mL). Chest radiography revealed no cardiomegaly or pulmonary congestion. Electrocardiography revealed sinus tachycardia (115 bpm) with nonspecific ST-T wave changes. Abdominal computed tomography (CT) showed right hydronephrosis, bilateral kidney stones, right urinary tract stones, right kidney swelling, and perirenal fat stranding (Figure 1). Echocardiography demonstrated a preserved LVEF (60%) and inspiratory collapse of the inferior vena cava.

**Table 1:** Laboratory data AST, aspartate aminotransferase; ALT, alanine aminotransferase; LDH, lactate dehydrogenase; Alb, albumin; UN, urea nitrogen; Cre, creatinine; NT-pro BNP, N-terminal prohormone of brain natriuretic peptide.

Laboratory data		
Laboratory examinations		Normal range
White blood cell count (/μL)	21,800	3,300-8,600
Hemoglobin (g/dL)	12.6	11.6-14.8
Hematocrit (%)	38.9	35.1-44.4
AST (U/L)	16	13-30
ALT (U/L)	10	7-23
LDH (U/L)	238	124-222
Alb (g/dL)	3.5	4.1-5.1
Serum UN (mg/dL)	24	8-20
Serum Cre (mg/dL)	1.16	0.46-0.79
Serum sodium (mEq/L)	143	138-145
Serum potassium (mEq/L)	3.3	3.6-4.8
C-reactive protein (mg/dL)	2.97	0-0.14
Procalcitonin (ng/mL)	2.04	0-0.5
NT-pro BNP (pg/mL)	654	0-125
Arterial blood gas analysis		
pH	7.46	
Lactate (mmol/L)	2.4	0-2.0
Blood and urine cultures		
Escherichia coli	+	

**Figure 2:**
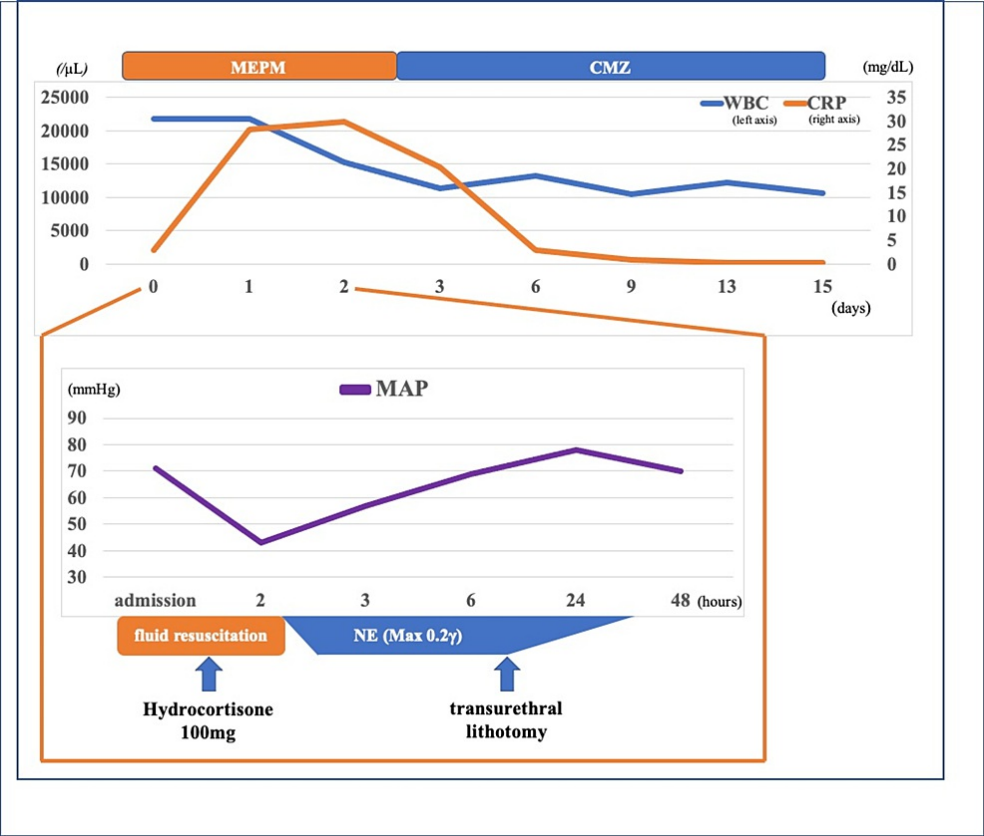
Treatment progress chart The upper panel shows antibiotic therapy and laboratory examinations. The lower panel shows the progress of treatment until the second day after hospitalization. CRP, C-reactive protein; CMZ, cefmetazole; MAP, mean arterial pressure; MEPM, meropenem; NE, norepinephrine; WBC, white blood cell.

**Figure 3:**
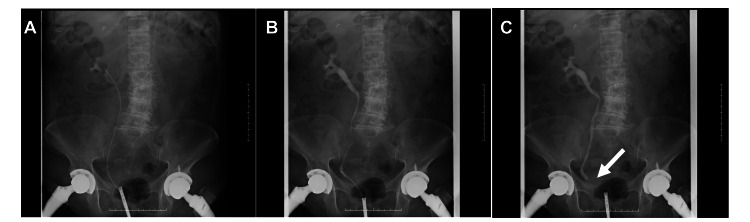
X-rays showing retrograde pyelogram (A) After removing the stones in the right urinary tract, the urinary catheter was advanced to the right renal pelvis. (B) The X-ray demonstrates the renal pelvis and right urinary tract. (C) Retrograde pyelogram after removal of the right ureteral stones showing temporary obstruction of the ureter (arrow).

One hour after transfer to our hospital, the patient developed hypotension (66/32 mmHg) and was diagnosed with septic shock due to UTI. Although she was given a bolus of 500 mL normal saline and 1,000 mL balanced crystalloids at a dose equivalent to 25 mL/kg, her blood pressure was not increased. Therefore, she was administered norepinephrine (NE, 0-0.2 μg/kg/min) and meropenem for sepsis (Figure 2). Hydrocortisone was administered immediately because of chronic steroid use and acute metabolic stress. CT confirmed obstruction of right urinary outflow due to kidney stones and urinary tract stones, and she underwent emergent transurethral lithotomy for her right urinary tract stones (Figure 3). Subsequently, the patient’s hemodynamics stabilized. On day 2, her SOFA score improved to 2. The dose of NE was gradually reduced and eventually discontinued with the resolution of her hemodynamic instability, although her CRP level was still elevated to 28.17 mg/dL. On day 4, the antibiotic was de-escalated to cefmetazole. On day 15, antibiotic therapy was terminated because the two sets of blood cultures collected after antibiotic therapy were negative. On day 16, her CRP level decreased to 0.21 mg/dL and she was discharged on day 17. Ten months after discharge, the patient had no recurrence of UTI or exacerbation of heart failure without dapagliflozin administration.

## Discussion

SGLT2 inhibitors are recommended as class I medicines for the treatment of patients with heart failure and reduced ejection fraction [1]. In a recent study, dapagliflozin significantly improved symptoms, physical limitations, and objectively measured exercise function in patients with heart failure with a preserved ejection fraction [2]. Our patient complained of shortness of breath during exertion in the outpatient department and was prescribed dapagliflozin because of an ejection fraction of 36%.

Several adverse effects of SGLT2 inhibitors have been reported, including acute kidney injury, ketoacidosis, and UTIs [3-5]. However, Donnan et al. reported that SGLT2 inhibitors did not increase these adverse events in their meta-analyses of randomized controlled trials [6]. In their subgroup analysis of individual SGLT2 inhibitors, dapagliflozin appeared to independently increase the risk of UTIs, although the biological mechanisms were unknown.

The risk factors for UTIs in patients treated with SGLT2 inhibitors are CKD, mood disorders, female sex, and older age [7-9]. Our patient was a female patient with CKD. Moreover, she was an immunosuppressed patient prescribed 14 mg of prednisolone and 1 mg of tacrolimus for 33 years and had urinary tract stones. As bacterial infections are increased by the quantity of immunosuppressive drugs, our patient was more likely to be infected than non-immunocompromised patients [10]. Therefore, she was considered a high-risk patient who developed a UTI because of dapagliflozin administration. When prescribing SGLT2 inhibitors to patients, clinicians should always consider the possibility of UTIs and cases in which the drugs should be used. In particular, if a patient is female and immunocompromised, dapagliflozin should be prescribed more carefully after considering the increased risk of UTIs.

## Conclusions

Although SGLT2 inhibitors are administered worldwide to patients with heart failure, they may induce urinary tract infections (UTIs) with septic shock. If the patient is considered to have a high risk of developing UTIs due to SGLT2 inhibitors like our patient, clinicians should accurately assess the risk of UTIs and reconsider the need for prescribing.
